# Supplementary material for: Conditional chemoconnectomics (cCCTomics) as a strategy for efficient and conditional targeting of chemical transmission
Source: eLife. 2024 Apr 30;12:RP91927. doi: 10.7554/eLife.91927 (PMC11060718; doi:10.7554/eLife.91927)
Supplement: Figure 3—source data 3. [file elife-91927-fig3-data3.zip › Figure 3 source data3 part 2.zip!/Figure 3-Source data 3-part2/1053-1-CRISPResso_Report_mpeqg9da.zip!/CRISPResso_on_1053_1/1a.Read_barplot.pdf]

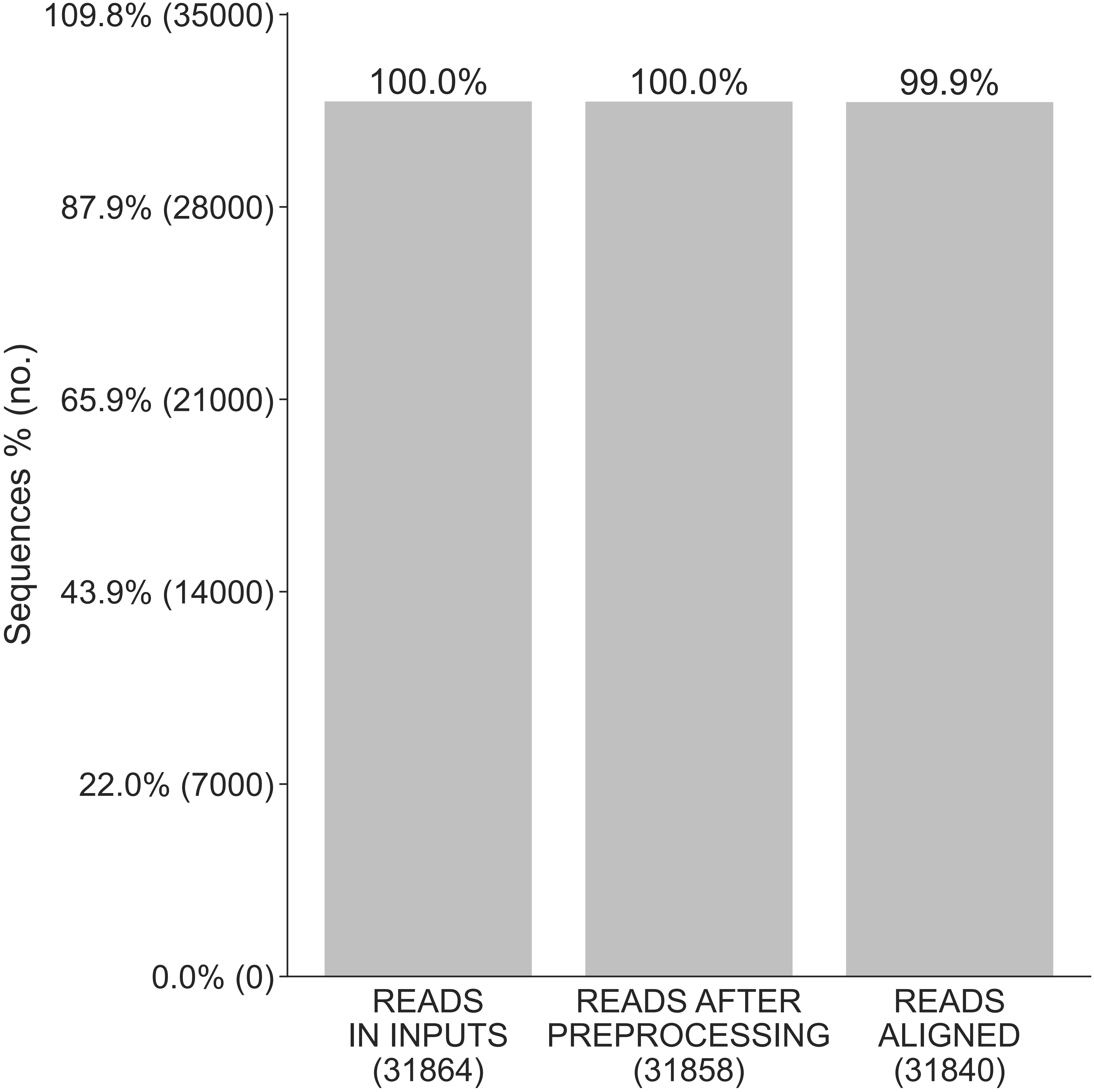

Sequences % (no.)
109.8% (35000)
87.9% (28000)
65.9% (21000)
43.9% (14000)
22.0% (7000)
0.0% (0)
100.0%
100.0%
99.9%
READS IN INPUTS (31864)
READS AFTER PREPROCESSING (31858)
READS ALIGNED (31840)
